# Addressing Systemic Racism by Creating a Diversity, Equity, and Inclusion Department Initiative

**DOI:** 10.1093/geroni/igab046.2090

**Published:** 2021-12-17

**Authors:** Stephanie Chow, Katherine Brown, Martine Sanon, Sasha Perez, Amy Kelley, Noelle Marie Javier

**Affiliations:** 1 Icahn School of Medicine at Mount Sinai, New York, New York, United States; 2 Icahn School of Medicine at Mount Sinai Hospital, Icahn School of Medicine at Mount Sinai, New York, United States

## Abstract

**Background:**

Catalyzed by social injustice and worsening racial inequities highlighted by the COVID-19 pandemic, a diverse academic geriatrics and palliative medicine department in NYC launched a DEI initiative. This report presents key program components and lessons learned in launching this initiative in the interprofessional academic medicine setting.

**Methods:**

First, DEI core and departmental administration met 2-4 times/month to plan and review program activities, vision, and mission. The team conducted confidential roundtable discussions about DEI issues and 1:1 interviews to assess needs. A monthly Humanities, Arts, and Books (HAB) Initiative provided a safe space for discussion and learning. The HAB platform supported a longitudinal curriculum emphasizing (1) group discussion and self-reflection on DEI concerns, (2) knowledge dissemination including a “Learning Pathway” series, and (3) skill-based workshops. With each event, we collected anonymous feedback. Comments were systematically recorded and an engagement evaluation was conducted to iteratively shape future sessions. Departmental administration was engaged to track DEI-focused measures of recruitment, career advancement, and retention. Finally, we centralized DEI activities on a departmental website, including an anonymous online feedback box.

**Results:**

Quantitative and qualitative assessment of DEI initiatives are forthcoming. Metrics include DEI and professional development surveys, departmental demographic and diversity measures, increase in DEI-related projects and grants, and individual participation in DEI programs.

**Conclusions:**

Creating a strong and sustainable DEI initiative within an academic medical setting requires a passionate and diverse core team, deliberate backing by administration, and thoughtful dissemination of sensitive content in the midst of a highly charged social justice landscape.

